# Supplementary material for: Perspectives of key stakeholders on integrating wearable sensor technology into rehabilitation care: a mixed-methods analysis
Source: Front Digit Health. 2025 Apr 28;7:1534419. doi: 10.3389/fdgth.2025.1534419 (PMC12066443; doi:10.3389/fdgth.2025.1534419)
Supplement: Supplementary file 1 [file Table1.docx]

| **Research Question** | **Themes** |
| --- | --- |
| 1. How do clinicians perceive the constructs of activity capacity and activity performance and their relationship in outpatient rehabilitation? | - Clinician perspectives on constructs of activity capacity and activity performance |
| 1. How do clinicians and patients perceive the value of activity performance monitoring in outpatient rehabilitation care? | - Patient perspectives on activity performance monitoring - Clinician perspectives on impact of activity performance monitoring on care - Clinician education/training |
| 1. What approaches, if any, are currently being used to measure activity performance in daily life in outpatient rehabilitation practice and what are important considerations regarding these approaches? | - Cost considerations to purchase a wearable sensor - Clinician knowledge/readiness to change - Comfort with technology (on the part of the clinician or patient) - Patient impairments that impact adherence with remote performance monitoring - Trust in the accuracy of consumer-grade wearable sensors to measure activity performance in patients - Ease of device use and ability to view the data |
| 1. What are the data collection and workflow preferences of clinicians and patients for integrating wearable sensor technology into rehabilitation care? | - Suggestions to support implementation/workflow integration of activity performance monitoring in care - Electronic health record preferences |

Supplemental Table 1. Final List of Themes for Each Research Question
